# Trends of poisoning types in Sri Lanka: descriptive analysis of hospital admissions data 2004–2019

**DOI:** 10.1186/s12889-025-24349-w

**Published:** 2025-11-14

**Authors:** Bruna Rubbo, Thilini Rajapakse, Lalith Senarathna, Janaka Pushpakumara, Michael Eddleston, Chris Metcalfe, Duleeka Knipe

**Affiliations:** 1 Population Health Sciences, Bristol Medical School, University of Bristol, Bristol, UK; 2 Centre for Pesticide Suicide Prevention and Centre for Cardiovascular Science, University of Edinburgh, Edinburgh, UK; 3 Department of Psychiatry, Faculty of Medicine, University of Peradeniya, Peradeniya, Sri Lanka; 4 South Asian Clinical Toxicology Research Collaboration, Faculty of Medicine, University of Peradeniya, Peradeniya, Sri Lanka; 5 Department of Health Promotion, Faculty of Applied Sciences, Rajarata University of Sri Lanka, Mihintale, Sri Lanka; 6 Faculty of Medicine and Allied Sciences, Rajarata University of Sri Lanka, Mihintale, Sri Lanka

**Keywords:** Pesticide poisoning, Case fatality, Ban, Medicinal poisoning, In-hospital deaths, Means restriction, Highly hazardous pesticides, Mortality, Public health intervention, Hospital admissions, Sri Lanka

## Abstract

**Background:**

Sri Lanka introduced national bans restricting access to highly hazardous pesticides (HHPs) in 2008-11, and in 2013-16. An immediate drop in pesticide poisoning cases was observed after the introduction of the first ban, but there was a simultaneous rise in hospital admissions due to drugs, medicines and biological substances. However, the long-term trends in hospital admissions and deaths due to poisoning in Sri Lanka have not been investigated. We aimed to determine whether there have been changes in types of poisoning presenting to hospitals and their associated case fatality (CF) following two distinct periods of implementation of national bans of HHPs in Sri Lanka.

**Methods:**

We conducted a retrospective observational study using routinely collected national-level hospital admissions data from Sri Lanka between 2004 and 2019 to examine trends in hospital admissions, deaths, and CF of different poisoning types. We included the following types: drugs, medicines and biological substances; pesticides; and non-medicinal products; and an additional group for other external causes. We calculated type-specific number of in-hospital cases and deaths per 100,000 population and annual CF, stratified by sex and age group.

**Results:**

We found a reduction in hospital admission cases from both pesticide poisoning (58.5% between 2012 and 2017) and drugs, medicine, and biological substances following the implementation of the first HHPs bans in 2011. There was an increase in hospital admissions due to non-medicinal products and other external causes, but this did not translate into an increase in hospital deaths or CF. We observed a sharp decrease in CF due to pesticide poisoning following the first ban (50% between 2008 and 2012), with no concurrent rise in CF due to other types of poisoning and non-poisoning substances, which remained low throughout the study period.

**Conclusions:**

The implementation of national bans of HHPs led to a reduction in number of admissions as well as CF due to pesticide poisoning in hospitals in Sri Lanka, with no evidence of substitution to other types of poisonings. The reversal of the upward trend in hospital admissions due to pesticide and medicinal poisonings suggests that the bans contributed to a reduction in admissions from these types of poisoning.

**Supplementary Information:**

The online version contains supplementary material available at 10.1186/s12889-025-24349-w.

## Background

Acute self-poisoning is one of the most frequently used methods of self-harm and suicide worldwide [1]. Pesticide poisoning is a common cause of in-hospital deaths in low- and middle-income countries (LMIC). Unlike in high-income countries (HIC), where most cases of pesticide poisoning are due to occupational or accidental exposures, most deaths in LMIC are attributed to intentional ingestion for self-harm (regardless of intent) [2–4]. Historically, pesticide self-poisoning was the most common method of suicide in Sri Lanka, but in more recent years deaths due to this method have fallen markedly (from 42 per 100,000 in 1994-96 to 11 per 100,000 by 2010-12), although it remains the second most common method of suicide in Sri Lanka [5, 6].

The availability of self-harm methods is directly associated with method choice, and with mortality rates through the lethality of the methods available [7]. In HIC, the most common method is by medicinal poisoning, often associated with low lethality (e.g. paracetamol case fatality (CF) < 0.5%) [8]. In Sri Lanka, the agricultural sector employs approximately a third of the population, and many families living in rural areas engage in farming [9]. This widescale agrarian activity, combined with heavy marketing of agrochemicals for farming, means that pesticides are both easily available and accessible in Sri Lanka. The high lethality of highly hazardous pesticides (HHPs) often means that acts of self-harm with no or low suicidal intent result in death.

Whilst hospital admissions for pesticide poisoning in Sri Lanka dropped following the introduction of a series of bans on HHPs aimed at restricting their access, there was a concomitant rise in admissions for poisoning by drugs, medicines and other biological substances (International Classification of Diseases (ICD)−10 categories T36-T50) [5, 10]. This rise has been of concern locally, as whilst fewer patients are dying and presenting with pesticide poisoning, the year-on-year rise of medicinal poisoning is a considerable burden for overstretched health services. As other countries consider and implement bans on HHPs in line with recommendation from the World Health Organization (WHO) for suicide prevention, they will be looking to countries, like Sri Lanka, where bans have been in place for a longer period of time to help them predict and plan their own services and prevention efforts [11]. Therefore, an updated investigation on hospital admission data will be important for both public health and health service planning in Sri Lanka and other countries.

Using routinely collected national level data, we aimed to determine whether there have been changes in type of substances used for poisoning. We also aimed to examine changes in CF for different types of poisoning following two distinct periods of implementation of national-level pesticide bans, of which the first was between 2008 and 2012, where paraquat, dimethoate, and fenthion were banned, and the second was between 2013 and 2016, where carbofuran 3% granules, carbaryl, chlorpyrifos, and glyphosate were banned.

## Methods

We conducted a retrospective observational study of routinely available panel data.

### Hospital admission data

We obtained the annual count, by age and sex, of all hospital admissions and deaths from 2004 to 2019 from the Annual Health Bulletins, published by the Sri Lankan Ministry of Health (available at https://www.health.gov.lk/moh_final/english/others.php?pid=110). These records are publicly available and contain data on causes of admission from all hospitals in Sri Lanka, classified according to ICD-10. We excluded data prior to 2004 as these were not stratified by age and sex.

We included records that were classified as poisoning, as follows: (i) poisoning by drugs, medicines and biological substances (T36-T50); (ii) toxic effects of organophosphate and carbamate insecticides (T60.0); (iii) toxic effects of other pesticides (T60.1-T60.9); and (iv) toxic effects of other, chiefly non-medicinal substances, including alcohol, solvents, halogen derivatives, corrosives, metals, gases, plants (including oleander seed), and food (including alcohol) (T51-T59, T61, T62, T63.1-T65). The full list of ICD-10 codes are available in supplementary table S1. We grouped (ii) and (iii) to form the ‘pesticides’ poisoning group. We included two additional categories: other unspecified effects of external causes (T33-T35, T66-T78); and sequelae of injuries, poisoning and of other consequences of external causes (T90-T98). These were grouped to form the ‘other external’ group, which we used as a comparator since pesticide poisoning cases and deaths can be misclassified as other external causes, and to explore whether there was any evidence of a potential change from poisoning to non-poisoning cases [12]. Previous research has shown that the majority of acute poisoning cases in some Sri Lankan regions are due to self-harm; however, we opted to refer to these cases as poisoning as it is unclear if these regional patterns can be generalized to the whole country [13–15].

Hospital admissions data were available by sex and age groups (17 to 49 years, 50 to 69 years, and 70 years and over). We excluded the following age groups as they were unlikely to contain cases and deaths of suicide by poisoning: less than 1 year, 1 to 4 years, and 5 to 16 years, and we were unable to ungroup the age category that included 5 to 16 year-olds [16].

### Population data

We obtained estimate mid-year populations from the Registrar General’s Department of Sri Lanka, and calculated annual rates (available at https://www.rgd.gov.lk/web/index.php/en/statistics2/vital-statistics.html). To smooth out step changes in population estimates that occurred around the Census years, we corrected the mid-year estimates as previously done [5]. Age was collapsed into the three groups described above to align with the hospital admissions data.

### Pesticide bans

In 2006, Sri Lanka introduced a mandatory reduction in the concentration of paraquat from 20 to 6.5%, which was followed by implementation of a 3-year phased ban in 2009. Paraquat was widely used as an herbicide in Sri Lanka and had a reported CF of 42% in its original 20% formulation [17]. Dimethoate and fenthion (CF 19% and 14%, respectively) were also banned between 2008 and 2010 [17]. Sri Lanka introduced bans on four other pesticides between 2013 and 2016, for reasons unrelated to acute toxicity (i.e. carbofuran 3% granules (CF 2%), carbaryl (7%), chlorpyrifos (CF 6%), and glyphosate (2%)) [17, 18].

Methods and results for pesticide usage data are available in the supplementary data.

### Analysis

We calculated annual number of cases and in-hospital deaths per 100,000 population, stratified by type of poison, sex, and age groups. Annual CF were obtained by dividing the number of deaths per number of cases for each of the ICD-10 causes of admission included, stratified by sex and age group.

We presented graphically the trends in poisoning, stratified by sex and age group. Data were linked, analysed, and plotted in R version 4.2.1, using the tidyverse, ggplot2, and tidylog packages.

## Results

### Hospital admission cases

**Fig. 1:**
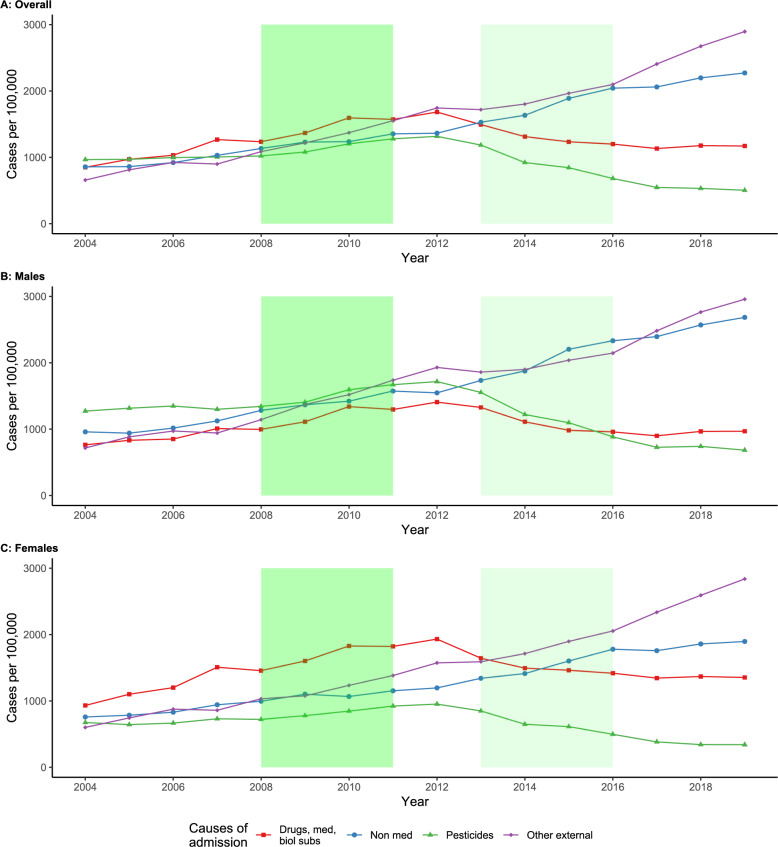
Number of hospital admission cases per 100,000 population, stratified by cause of admission and by sex Footnote: Shaded areas represent the 2008–2011 ban on highly hazardous pesticides (dark green) and the 2013–2016 ban on other pesticides (light green). Med: medicines; biol. subs.: biological substances

We found a steady increase in the number of pesticide poisoning cases admitted to hospital up to 2012, corresponding to a 36.3% increase from 966.1 hospital admissions in 2004 to 1,316.4 hospital admissions per 100,000 population in 2012 (Fig. 1A). This was followed by a 58.5% decrease to 546.5 hospital admissions per 100,000 population in 2017, with numbers of hospital admissions due to pesticide poisoning remaining stable throughout 2018 and 2019. A similar pattern was observed for cases admitted due to poisoning by drugs, medicines and biological substances. There was an upward trend in cases up to 2012, followed by a decline until a plateau was reached around 1,100 hospital admissions per 100,000 population in 2016. Conversely, we observed an upward trend in admissions due to toxic effects of other, chiefly non-medicinal substances and due to other external causes throughout the entire study period. The latter became the most common cause of hospital admissions from 2012 onwards.

When stratified by sex, the most common cause of hospital admissions due to poisoning in males was pesticides until 2010, when there were 1,593 hospital admissions per 100,000 males (Fig. 1B). Admissions due to pesticide poisoning was surpassed by other external causes in 2011. However, pesticide poisoning continued to rise until 2012, with a peak of 1,717 hospital admissions per 100,000 males, followed by a 60.2% reduction in number of admission cases by 2019 (683 per 100,000 males). In females, poisoning by drugs, medicines and biological substances was the most common cause of admission until 2014 (Fig. 1C). Pesticide poisoning was the least common cause of admission in females from 2005 onwards, but the pattern observed was similar to those in males, with a peak of 952.7 hospital admissions in 2012 followed by a 64.3% reduction to 340.4 per 100,000 females by 2019. In both sexes, there was an increase in hospital admission cases due to toxic effects of other, chiefly non-medicinal substances and due to other external causes, observed from 2004 to 2019 (Figure S1).

### Hospital deaths

**Fig. 2:**
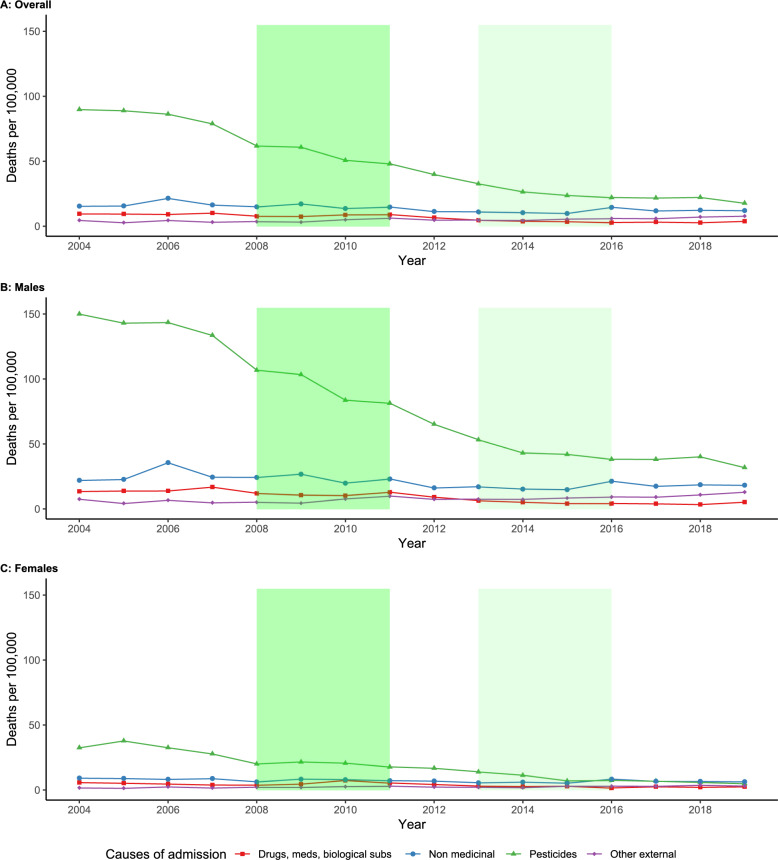
Number of in-hospital deaths per 100,000 population, stratified by cause of admission and by sex Footnote: Shaded areas represent the 2008–2011 ban on highly hazardous pesticides (dark green) and the 2013–2016 ban on other pesticides (light green). Med: medicines; biol. subs.: biological substances

The most common cause of in-hospital death due to poisoning in 2004 was pesticide poisoning (89.9 deaths per 100,000 population) (Fig. 2). Other causes were only responsible for a small proportion of cases (9.6 deaths per 100,000 due to drugs, medicines and biological substances; 15.5 deaths per 100,000 due to toxic effects of other, chiefly non-medicinal substances; and 4.6 deaths per 100,000 due to other external causes). Deaths by pesticide poisoning were considerably higher in males (150.1 deaths per 100,000 compared to 32.6 deaths per 100,000 in females in 2004). We observed a steep decrease in pesticide poisoning deaths in both sexes from 2004 to 2019 (78.7% decrease for males and 84.8% for females).

### Case fatalities

**Fig. 3:**
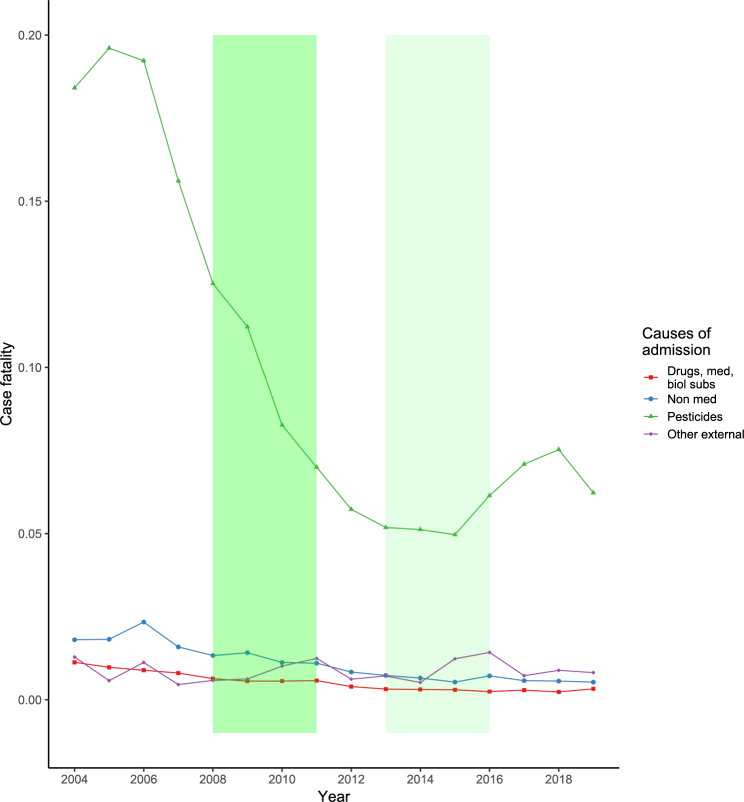
Overall case fatalities for different ICD-10 categories of poisoning Footnote: Shaded areas represent the 2008–2011 ban on highly hazardous pesticides (dark green) and the 2013–2016 ban on other pesticides (light green). Med: medicines; biol. subs.: biological substances

We found a marked reduction in CF from pesticide poisoning from 2007 onwards, which was observed in both sexes and across all age groups (Figs. 3). CF for other poisoning types remained low and stable throughout the study period, with similar results when stratified by sex and age group.

Overall pesticide poisoning CF was at its highest between 2004 and 2006 (range 0.18 to 0.20 deaths per cases). This was followed by a steep decrease, with overall pesticide poisoning CF halving between 2008 and 2012, following the phased ban on paraquat, dimethoate, and fenthion (2008–2011).

After the second pesticide bans, implemented between 2013 and 2016, we observed a trend shift, with an increase to a peak CF of 0.08 in 2018 (Fig. 3). However, this was a 60% reduction in CF from the peak CF observed in 2005. We found the same patterns when stratified by sex and age groups, particularly in younger males, and older males and females (Figure S2).

In males 70 years of age or older, the decline in CF was even more pronounced from 2008 onwards, when the phased ban of paraquat, dimethoate and fenthion was first introduced (from 0.92 in 2008 to 0.28 in 2012, a 69.6% reduction) (Figure S2 D). This downward trend continued until the introduction and implementation of the second ban on less acutely toxic pesticides. CF increased by 75% in this subpopulation, from 0.28 in 2012 to a peak of 0.49 in 2016. In females, pesticide poisoning CF was higher in older women compared to those in the 17 to 49 age group, particularly in recent years (Figure S2 E-H).

## Discussion

We showed that the increase in hospital admission cases of poisoning by drugs, medicines and biological substances, previously observed up to 2010, was not sustained beyond 2012 and the pattern of hospital admissions by age and sex for this type of poisoning was similar to that for pesticides [5, 10]. We found a considerable reduction in pesticide poisoning admissions, and these were not associated with concurrent rises in case fatalities due to other poisoning or non-poisoning methods between 2004 and 2019 in Sri Lanka. The CF for pesticide poisoning declined from 2007 onwards and were at their lowest following the implementation of the 3-year phased ban on paraquat, dimethoate, and fenthion in 2011. This reduction in CF due to pesticide poisoning was not offset by increases in deaths to other poisoning types. These findings suggest that limiting access to HHPs can be cost-effective, as the numbers of hospital admissions and deaths due to poisoning by both pesticides and drugs, medicines and biological substances decreased following the implementation of the national bans.

We observed an upward trend in hospital admissions due to toxic effects of other, chiefly non-medicinal substances, due to other unspecified effects of external causes, and due to sequelae of injuries, poisoning and of other consequences of external causes throughout the study period. Medications are widely regarded as healing products in Sri Lanka, while non-medicinal products such as plants (e.g. oleander seeds) and cleaning products, which are widely available in and around households, are more commonly viewed as poisonous when ingested. However, CF due to these methods remained low from 2004 to 2019, and hospital admissions, which were already rising prior to the bans, continued to increase at a similar pace after the bans.

Since individuals attempting self-harm and suicide tend to opt for methods they are most familiar with and have easy access to in moments of crisis, banning one pesticide could lead to switching to other available pesticides [19, 20]. With the removal of HHPs from the market in Sri Lanka, we would expect to see a reduction in the CF associated with poisoning, which we observed. If a switch from HHPs to less toxic pesticides that were still available following the bans occurred, we would expect to see a continuation in the trend of admissions due to pesticide poisoning, as previously observed in Sri Lanka [21]. However, we found a decrease in hospital admissions due to this type of poisoning. The reasons for this decrease are not clear. It may be due to higher cost of the pesticides that were introduced as alternatives following the bans on HHPs, which might have led to fewer pesticides being bought and stored in households, limiting availability and access to these poisons. There could have been a switch from pesticide ingestion to methods not captured by hospital data, such as suicide by hanging. Studies have suggested that means restriction could lead to method substitution, with other highly lethal methods becoming more frequent. This has been raised as a concern in LMIC following pesticide bans, including Sri Lanka and India [5, 20, 22–26].

Evidence from several LMICs where bans on pesticides have been implemented suggests that methods restriction can prevent suicide [27]. For example, in Malaysia, deaths from hospital admissions by paraquat poisoning decreased by 12% and the case fatality fell by 4% following a phased paraquat ban [28]. In Bangladesh, reductions in hospital mortality due to pesticide suicides were associated with bans on HHPs, despite an observed increase in pesticide use [29]. The cumulative effect of a series of bans on pesticides implemented in Sri Lanka between 1984 and 2012 led to the prevention of an estimated 93,000 suicide deaths [30]. In HIC, the picture is less clear, with some countries reporting a small reduction in pesticide suicides following bans [27, 31]. This is likely because the proportion of suicides due to pesticide ingestion is smaller in HIC compared to LMIC.

Since 2016 the rate of suicide in Sri Lankan males has started to increase, largely driven by a rise in suicide deaths by hanging [32–34]. Following the time of the implementation of the bans the most frequently mentioned method of suicide in Sri Lankan newspapers between 2014 and 2015 was by hanging; research has shown that this may lead to an uptake of this method, particularly by vulnerable individuals which may explain the rise in suicide deaths by this method. Data on suicide deaths that occur in any setting in Sri Lanka are only captured within suicide statistics collected by police departments so future analysis of the national suicide data needs to explore the possibility of method substitution in more recent years.

We found a steeper decrease in CF for pesticide poisoning in older men; however, CF continued to be higher than in younger individuals. This higher CF in older men is in line with findings from South Korea and China and could reflect a worse prognosis following pesticide poisoning in the elderly due to comorbidities or higher suicidal intent [3, 23, 24, 26, 35]. The marked decline in CF in older men in Sri Lanka is consistent with age- and sex-stratified suicide data from previous research, which showed that pesticide poisoning is more common in this subpopulation [3].

We observed a slight upward trend in CF after the implementation of the second ban on pesticides, from 2016 onwards. This could signal a shift from less acutely toxic pesticides such as chlorpyrifos and glyphosate, which have CFs of 6% and 2% respectively, to other more toxic pesticides that were still available in Sri Lanka [16]. Research from rural Sri Lanka revealed an increase in the use of profenofos and carbosulfan for suicide from 2014 onwards [17, 36]. This likely would not have occurred if chlorpyrifos, which has relatively low toxicity, had not been banned. However, we found a considerable drop in CF (60% decrease) from the peak CF in the years preceding the first ban to the highest rate in 2018, after implementation of the second ban.

Our investigation has several strengths. We utilised a routinely collected national dataset on hospital admission covering a period of 16 years. We explored overall trends by type of poisoning, as well as by sex and age group. These kinds of data are rare for LMIC. Despite its strengths, the findings of the study must be interpreted in light of its limitations. Firstly, trends in poisoning rates might have been affected by other internal and external factors such as improvement in healthcare delivery and hospital resources, establishment of national poisoning centres, economic crisis, and armed conflicts, as our data cover a long period of time. However, studies have found that previous bans on WHO class I pesticides in Sri Lanka led to a decline in suicide rates, independent of secular trend in other risk factors such as unemployment, alcohol abuse, divorce, or civil war [27, 37]. Secondly, the poisoning groups are not ideal categories to explore self-harm poisoning as it includes non-self-harm cases. However, in the case of pesticide poisoning, most cases in Sri Lanka are due to self-harm [13, 38]. Thirdly, we were limited by the age groups that were available. Due to the lack of more granular data, we were not able to investigate poisoning trends in those younger than 17 years of age or in smaller age intervals. Since age data are collected and recorded as a continuous variable, we strongly recommend that the ungrouped data be made available on centralised databases. Additionally, we were unable to align these age groups with those available in the population data. This is particularly important as previous research from rural areas in Sri Lanka has shown that the largest change in pesticide poisoning occurred in females between 15 and 19 years of age [39]. Whilst exclusion of individuals under 17 years of age is a limitation, the number of suicide deaths in this age group are small and therefore unlikely to unduly impact on the overall conclusions of this study [16]. Fourthly, since we only used hospital admissions data, we were unable to investigate out-of-hospital deaths, such as hanging, as these are captured by police data. Lastly, since organophosphorus and carbamate insecticides were grouped in the original dataset, we were unable to distinguish between admissions and deaths by specific types of pesticides. We were also not able to exclude some of the ICD-10 codes that were less relevant to our study, as these were only available at the aggregate level.

## Conclusions

We show that the implementation of national pesticide bans led to a reduction in CF due to pesticide poisoning in hospitals in Sri Lanka, with no evidence of substitution from other types of poisonings. The availability of less lethal poisons instead of HHPs can prevent fatal cases from occurring and enable appropriate social and psychological support to be provided to avoid deaths. Our findings have public health implications, as they highlight the effectiveness of means restriction in reducing hospital admissions and deaths due to pesticide poisoning. The reversal of the upward trend in pesticide and medicinal poisoning suggests that the adoption and implementation of bans may be an appropriate method to reduce the number of hospital admissions from poisoning, potentially preserving important hospital resources, which are often scarce in LMIC settings. However, formal health economic evaluation studies are needed in order to further explore these findings. The increase in hospital admissions due to non-medicinal products and other external causes was not related to the implementation of bans on HHPs, and did not translate into an increase in hospital deaths or CF. However, further research on method-specific suicides combining hospital, community, and death data at the national and regional level, as well as qualitative interview data from survivors of self-harm to explore the occurrence of method substitution is needed in Sri Lanka.

## Supplementary Information

Supplementary Material 1.

## Data Availability

The datasets used in the current study are available from the links provided in the methods section of the manuscript.

## Abbreviations

CF: Case fatality
HHP: Highly hazardous pesticides
LMIC: Low- and middle-income countries
HIC: High-income countries
ICD: International Classification of Diseases
WHO: World Health Organization
