# Supplementary material for: Trends of poisoning types in Sri Lanka: descriptive analysis of hospital admissions data 2004–2019
Source: BMC Public Health. 2025 Nov 14;25:3959. doi: 10.1186/s12889-025-24349-w (PMC12619267; doi:10.1186/s12889-025-24349-w)

# Supplementary materials

# Methods

## Pesticide usage data

We obtained pesticide sales data on imports and exports of total pesticides (in tons) from 2004 to 2019 from the Food and Agriculture Organisation (FAO) of the United Nations. These data are collected using standardised questionnaires, completed with official government data sources (e.g. ministerial data portals), and are publicly available at <http://www.fao.org/faostat/en/#data/RP>. Missing values were imputed by the FAO for the years of 2013 and 2016 to 2018.

# Results

## Pesticide import and export data

We observed a decrease in the total import of pesticides in Sri Lanka from 2011 onwards (Figure S3). Volume of pesticide imports was 10,120 tons in 2008, preceding the 3-year phased ban on Paraquat. There was a slight decrease in pesticide imports in 2009 when the phased based was initiated, followed by a sharp increase in 2010, peaking at 16,549 tons. Pesticide imports started to decline when the 3-year paraquat ban was fully implemented, reaching a low of 5,515 tons in 2017.

**Table S1.** List of ICD-10 codes included in this study.

| **ICD-10 category** | **Label** |
| --- | --- |
| T36 - T50 | Poisoning by drugs, medicaments and biological substances |
| T36 | Poisoning by systemic antibiotics |
| T37 | Poisoning by other systemic anti-infectives and antiparasitics |
| T38 | Poisoning by hormones and their synthetic substitutes and antagonists,  not elsewhere classified |
| T39 | Poisoning by nonopioid analgesics, antipyretics and antirheumatics |
| T40 | Poisoning by narcotics and psychodysleptics [hallucinogens] |
| T41 | Poisoning by anaesthetics and therapeutic gases |
| T42 | Poisoning by antiepileptic, sedative-hypnotic and antiparkinsonism drugs |
| T43 | Poisoning by psychotropic drugs, not elsewhere classified |
| T44 | Poisoning by drugs primarily affecting the autonomic nervous system |
| T45 | Poisoning by primarily systemic and haematological agents,  not elsewhere classified |
| T46 | Poisoning by agents primarily affecting the cardiovascular system |
| T47 | Poisoning by agents primarily affecting the gastrointestinal system |
| T48 | Poisoning by agents primarily acting on smooth and skeletal muscles  and the respiratory system |
| T49 | Poisoning by topical agents primarily affecting skin and mucous membrane  and by ophthalmological, otorhinolaryngological and dental drugs |
| T50 | Poisoning by diuretics and other and unspecified drugs, medicaments  and biological substances |
| T60 | Toxic effect of pesticides |
| **T60.0** | **Toxic effect of organophosphate and carbamate insecticides** |
| **T60.1-T60.9** | **Toxic effects of other pesticides** |
| T60.1 | Toxic effect of halogenated insecticides |
| T60.2 | Toxic effect of other and unspecified insecticides |
| T60.3 | Toxic effect of herbicides and fungicides |
| T60.4 | Toxic effect of rodenticides |
| T60.8 | Toxic effect of other pesticides |
| T60.9 | Toxic effect of pesticide, unspecified |
| **T51-T59, T61, T62, T63.1-T65** | **Toxic effects of other, chiefly non-medicinal substances** |
| T51 | Toxic effect of alcohol |
| T52 | Toxic effect of organic solvents |
| T53 | Toxic effect of halogen derivatives of aliphatic and aromatic hydrocarbons |
| T54 | Toxic effect of corrosive substances |
| T55 | Toxic effect of soaps and detergents |
| T56 | Toxic effect of metals |
| T57 | Toxic effect of other inorganic substances |
| T58 | Toxic effect of carbon monoxide |
| T59 | Toxic effect of other gases, fumes and vapours |
| T61 | Toxic effect of noxious substances eaten as seafood |
| T62 | Toxic effect of other noxious substances eaten as food |
| T63 | Toxic effect of contact with venomous animals |
| T64 | Toxic effect of aflatoxin and other mycotoxin food contaminants |
| T65 | Toxic effect of other and unspecified substances |
| **T33-T35, T66-T78** | **Other unspecified effects of external causes** |
| T33 | Superficial frostbite |
| T34 | Frostbite with tissue necrosis |
| T35 | Frostbite involving multiple body regions and unspecified frostbite |
| T66 | Unspecified effects of radiation |
| T67 | Effects of heat and light |
| T68 | Hypothermia |
| T69 | Other effects of reduced temperature |
| T70 | Effects of air pressure and water pressure |
| T71 | Asphyxiation |
| T73 | Effects of other deprivation |
| T74 | Maltreatment syndromes |
| T75 | Effects of other external causes (e.g. drowning and nonfatal submersion) |
| T76 | Unspecified effects of external causes (e.g. intentional self-harm (suicide)  by unspecified means) |
| T78 | Adverse effects, not elsewhere classified |
| **T90-T98** | **Sequelae of injuries, poisoning and of other consequences of external causes** |
| T90 | Sequelae of injuries of head |
| T91 | Sequelae of injuries of neck and trunk |
| T92 | Sequelae of injuries of upper limb |
| T93 | Sequelae of injuries of lower limb |
| T94 | Sequelae of injuries involving multiple and unspecified body regions |
| T95 | Sequelae of burns, corrosions and frostbite |
| T96 | Sequelae of poisoning by drugs, medicaments and biological substances |
| T97 | Sequelae of toxic effects of substances chiefly nonmedicinal as to source |
| T98 | Sequelae of other and unspecified effects of external causes |

**Table S2.** Number of hospital admission cases and deaths per 100,000 population, by year and cause of admission.

| Year | Admission cases per 100,000 population | | | | Deaths per 100,000 population | | | |
| --- | --- | --- | --- | --- | --- | --- | --- | --- |
|  | Drugs, med., biol. subs. | Non med. | Pesticides | Other external | Drugs, med., biol. subs. | Non med. | Pesticides | Other external |
| 2004 | 850.0 | 856.4 | 966.1 | 657.5 | 9.6 | 15.5 | 89.9 | 4.6 |
| 2005 | 970.3 | 860.1 | 970.1 | 811.8 | 9.5 | 15.7 | 89.0 | 2.8 |
| 2006 | 1031.4 | 920.6 | 996.5 | 922.6 | 9.2 | 21.5 | 86.3 | 4.5 |
| 2007 | 1267.1 | 1029.9 | 1005.2 | 899.2 | 10.2 | 16.4 | 78.9 | 3.2 |
| 2008 | 1235.2 | 1133.4 | 1020.6 | 1085.7 | 7.9 | 15.1 | 61.9 | 3.8 |
| 2009 | 1366.4 | 1228.6 | 1079.1 | 1217.4 | 7.7 | 17.4 | 61.0 | 3.4 |
| 2010 | 1594.6 | 1236.0 | 1203.3 | 1370.5 | 9.0 | 13.9 | 51.0 | 5.3 |
| 2011 | 1571.7 | 1353.3 | 1278.4 | 1551.4 | 9.1 | 14.9 | 48.2 | 6.4 |
| 2012 | 1682.1 | 1363.3 | 1316.4 | 1743.4 | 6.7 | 11.4 | 39.9 | 4.8 |
| 2013 | 1492.9 | 1528.4 | 1184.7 | 1718.3 | 4.8 | 11.2 | 32.9 | 4.9 |
| 2014 | 1311.6 | 1633.2 | 921.1 | 1801.7 | 4.1 | 10.7 | 26.7 | 4.7 |
| 2015 | 1233.6 | 1888.3 | 843.3 | 1964.3 | 3.7 | 10.1 | 23.9 | 5.8 |
| 2016 | 1199.3 | 2042.4 | 681.5 | 2097.5 | 3.0 | 14.7 | 22.2 | 6.1 |
| 2017 | 1132.4 | 2060.8 | 546.5 | 2406.0 | 3.3 | 11.9 | 21.8 | 5.9 |
| 2018 | 1176.5 | 2197.3 | 532.0 | 2673.8 | 2.8 | 12.4 | 22.3 | 7.2 |
| 2019 | 1170.4 | 2271.4 | 503.6 | 2895.1 | 3.8 | 12.0 | 17.8 | 7.8 |

**Figure S1.** Number of cases admitted to hospital per 100,000 sex- and age-specific population.


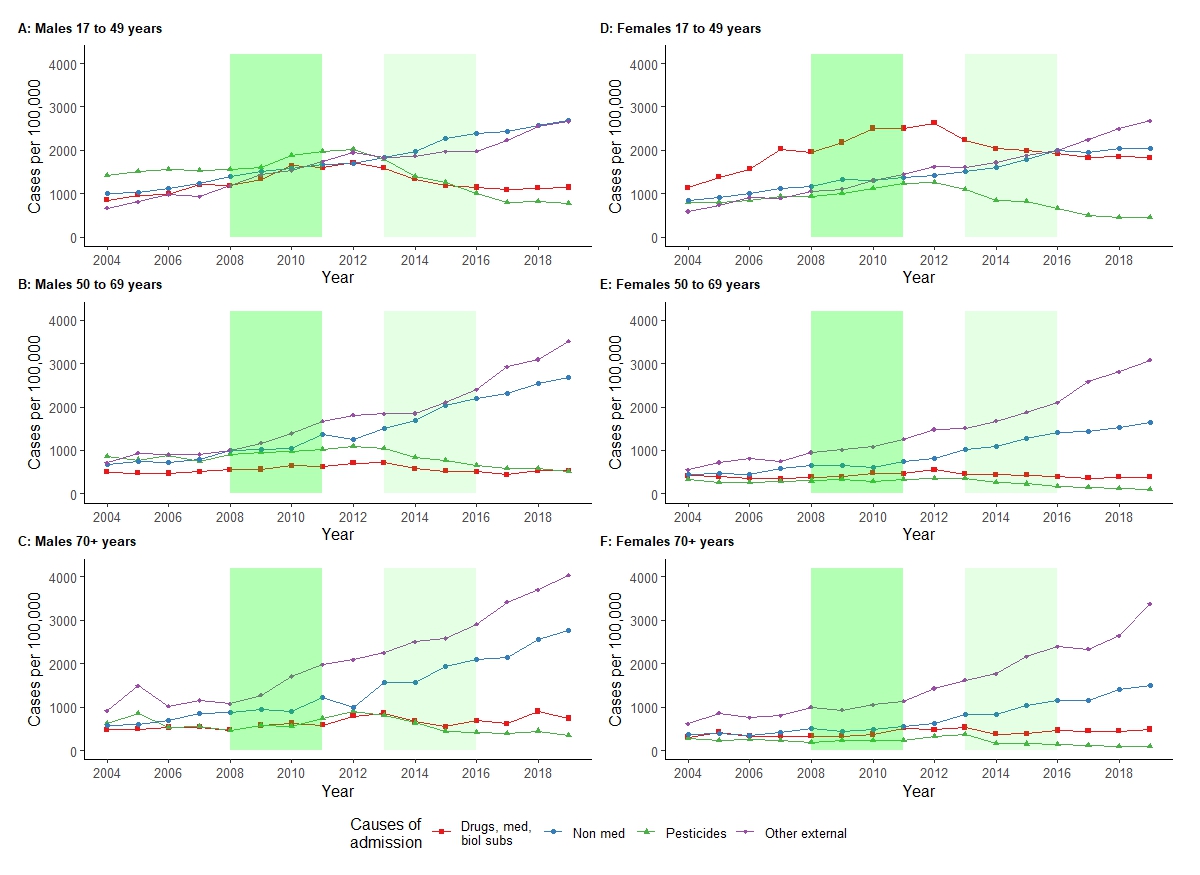


Shaded areas represent the 2008-2011 ban on highly hazardous pesticides (dark green) and the 2013-2016 ban on other pesticides (light green). Med: medicines; biol. subs.: biological substances.

**Figure S2.** Case fatalities for different categories of poisoning, stratified by sex and age group.


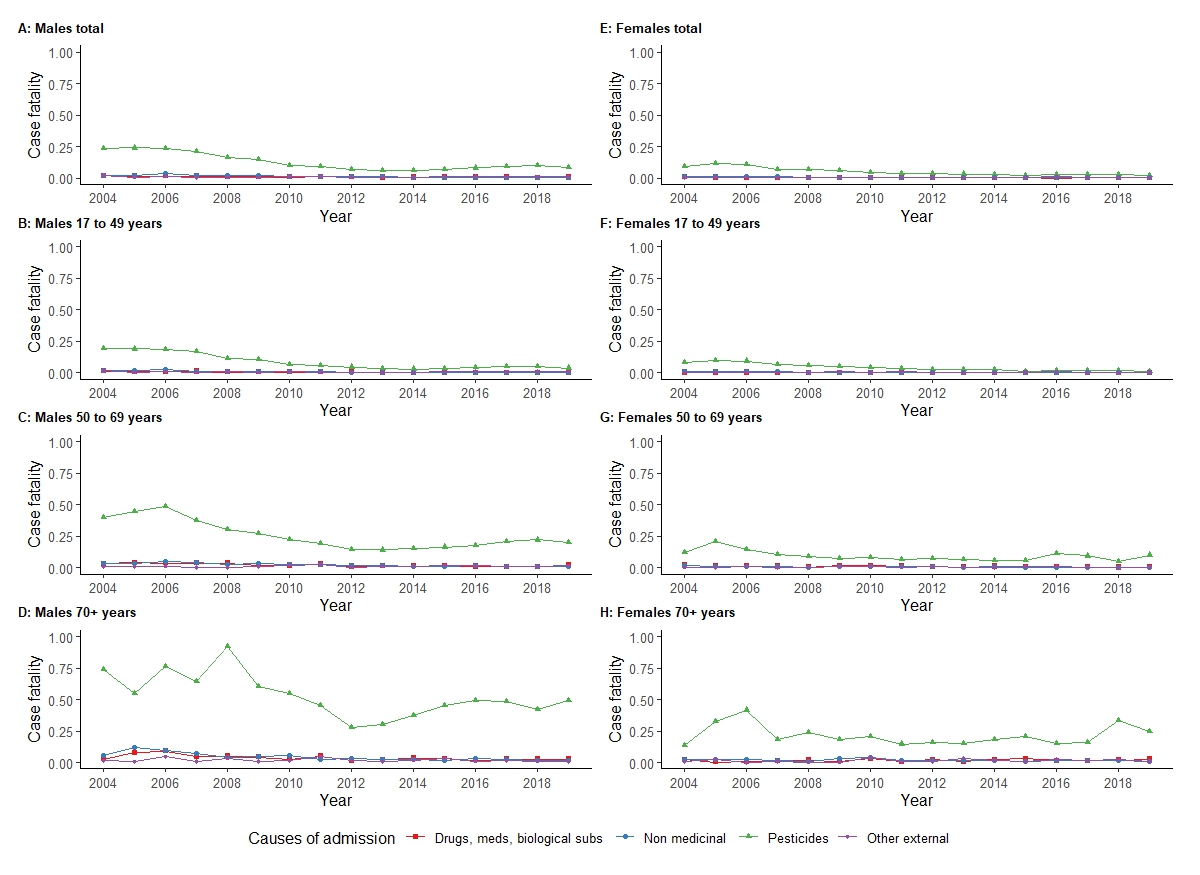


Shaded areas represent the 2008-2011 ban on highly hazardous pesticides (dark green) and the 2013-2016 ban on other pesticides (light green). Med: medicines; biol. subs.: biological substances.

**Figure S3.** Volume of pesticides imported into and exported from Sri Lanka during 2004 to 2019, according to the Food and Agriculture Organization of the United Nations.


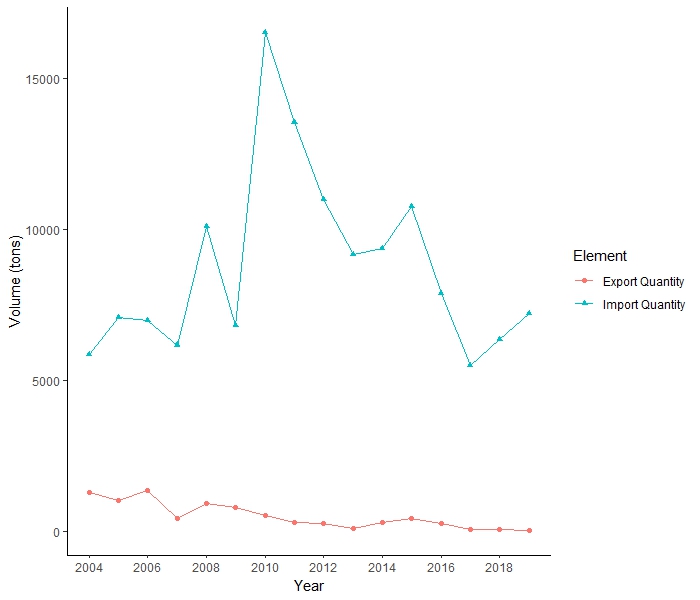

Supplement: Supplementary file 1 — Supplementary Material 1. [file 12889_2025_24349_MOESM1_ESM.docx]
